# Environmental pleiotropy and demographic history direct adaptation under antibiotic selection

**DOI:** 10.1038/s41437-018-0137-3

**Published:** 2018-09-06

**Authors:** Danna R. Gifford, Rok Krašovec, Elizabeth Aston, Roman V. Belavkin, Alastair Channon, Christopher G. Knight

**Affiliations:** 1 School of Biological Sciences, Faculty of Biology, Medicine and Health, The University of Manchester, Manchester, UK; 2 School of Earth and Environmental Sciences, Faculty of Science and Engineering, The University of Manchester, Manchester, UK; 3 School of Computing and Mathematics, Faculty of Natural Sciences, Keele University, Keele, UK; 4 School of Science and Technology, Middlesex University, London, UK

**Keywords:** Experimental evolution, Bacterial evolution

## Abstract

Evolutionary rescue following environmental change requires mutations permitting population growth in the new environment. If change is severe enough to prevent most of the population reproducing, rescue becomes reliant on mutations already present. If change is sustained, the fitness effects in both environments, and how they are associated—termed ‘environmental pleiotropy’—may determine which alleles are ultimately favoured. A population’s demographic history—its size over time—influences the variation present. Although demographic history is known to affect the probability of evolutionary rescue, how it interacts with environmental pleiotropy during severe and sustained environmental change remains unexplored. Here, we demonstrate how these factors interact during antibiotic resistance evolution, a key example of evolutionary rescue fuelled by pre-existing mutations with pleiotropic fitness effects. We combine published data with novel simulations to characterise environmental pleiotropy and its effects on resistance evolution under different demographic histories. Comparisons among resistance alleles typically revealed no correlation for fitness—i.e., neutral pleiotropy—above and below the sensitive strain’s minimum inhibitory concentration. Resistance allele frequency following experimental evolution showed opposing correlations with their fitness effects in the presence and absence of antibiotic. Simulations demonstrated that effects of environmental pleiotropy on allele frequencies depended on demographic history. At the population level, the major influence of environmental pleiotropy was on mean fitness, rather than the probability of evolutionary rescue or diversity. Our work suggests that determining both environmental pleiotropy and demographic history is critical for predicting resistance evolution, and we discuss the practicalities of this during in vivo evolution.

## Introduction

Genetic variation allows populations to evolve in response to natural selection. Adaptation in large, viable populations tends to proceed via large-effect beneficial mutations, as growth allows repeated sampling across the span of possible fitness effects Rozen et al. (2008). However, sudden environmental changes can produce conditions that drastically reduce reproduction, thereby limiting the generation of allelic diversity on which selection can act (Iwasa et al. 2004; Martin et al. 2013; Bell 2013; Gonzalez and Bell 2013; Bell 2017). The continuing survival of such populations is therefore predicated on the presence of key mutations in the population before environmental change occurs (Bell 2013; Orr and Unckless 2014). The availability of such mutations is dependent on demographic history, that is temporal fluctuations in population size (Kimura 1983, Charlesworth 2009). If selectively neutral prior to environmental change, diversity of these mutations will be governed by differences in mutation rates at different loci (Foster et al. 2013). However, differential selection prior to environmental change will alter this composition. Consequently, while organismal fitness typically determines population adaptation, following sudden environmental change, demographic history, locus mutation rates, and fitness in the old environment may also have considerable influence.

Antibiotic treatment is an important example of a sudden environmental change that limits reproduction (e.g., Bell and Gonzalez 2009; Alexander et al. 2014; Ojala et al. 2014). Recent work has shown that fitness, genetic diversity, and their interaction influence the probability of evolutionary rescue under antibiotic selection (Couce et al. 2016; Wilson et al. 2017; Anciaux et al. 2018). For rescue to occur, resistance alleles must be present prior to treatment because the ancestor cannot reproduce—and consequently, cannot generate genetic diversity—in the presence of antibiotic. Resistance typically imposes a fitness cost in antibiotic-free environments, which is selected against in the absence of antibiotic (Vogwill and Maclean 2015; Melnyk et al. 2015; but *cf*. Lenormand et al. 2018). Populations evolving resistance may therefore be pulled in alternate directions by selection, first favouring mutations with negligible fitness costs in the absence of antibiotic, and second favouring large fitness benefits in the presence of antibiotic. However, mutations that possess one of these characteristics do not necessarily possess the other, leading to a potential conflict during evolution of resistance from an initially sensitive ancestor. The relationship between fitness effects of alleles in each environment, termed ‘environmental pleiotropy’, determines whether selection acts in the same direction in each environment. For ‘positive pleiotropy’, the fitnesses of alleles are positively correlated between environments. For ‘negative pleiotropy’, fitnesses are negatively correlated (i.e., best becomes worst), and for ‘neutral pleiotropy’, fitnesses are uncorrelated. In order for pleiotropy to have any influence, however, multiple alleles must be present in the population prior to environmental change. Allelic diversity is determined by demographic history, which for microbial populations is primarily influenced by fluctuations in population size over time and the number of generations elapsed prior to antibiotic treatment. Demographic history of a population will therefore influence not only the probability of evolutionary rescue, but also the fitness of populations under sudden and sustained antibiotic selection.

Here, we demonstrate that environmental pleiotropy and demographic history interact to influence antibiotic resistance allele frequencies following selection for resistance. To approach this issue, we reanalysed published data from short- and long-term resistance selection experiments. We complement this analysis with novel computer simulations of antibiotic resistance evolution. In the reanalysed data, the fitness of different resistance alleles was weakly correlated between the extremes of antibiotic concentrations; this provides no evidence for strong positive or negative environmental pleiotropy. Mutant allele frequency following sustained selection for resistance showed a negative relationship with fitness in the presence of antibiotic, and a positive relationship in the absence of antibiotic. Simulations allowed us to explore in depth the interaction of environmental pleiotropy with demography and locus mutation rate. Although each affects mutant allele frequencies following sudden and sustained environmental change, the nature of the effect depends on the interaction among them. For instance, the effect of pleiotropy on mutant allele frequencies was contingent on demographic history, such as population size (fluctuating through periodic population bottlenecks), and the number of generations spent in the absence of antibiotic. Given the inherent difficulty in determining both demographic history and fitness effects for non-experimental microbial populations, we discuss the implications of these interactions for predicting the course of antibiotic resistance evolution.

## Methods

### Correlations of fitness measured at different antibiotic concentrations

To investigate environmental pleiotropy, we compared published fitness data of resistant mutants measured across a range of antibiotic concentrations above and below the sensitive ancestor’s minimum inhibitory concentration (MIC). Data were available for *E. coli* K-12 resistant to either rifampicin, nalidixic acid, or trimethoprim (Lindsey et al. 2013; Harmand et al. 2016; Palmer et al. 2015), and for 8 species of *Pseudomonas* resistant to rifampicin (Vogwill et al. 2014 2016a, see Table S1). These antibiotics were chosen because they are the best characterised in terms of fitness effects over a range of antibiotic concentrations. Resistance to these antibiotics can arise via single nucleotide polymorphisms (SNPs): rifampicin resistance via mutations in beta subunit of RNA polymerase *rpoB*; nalidixic acid resistance via mutations in DNA gyrase subunit A, *gyrA*; and trimethoprim resistance via mutations in the coding or promoter regions of dihydrofolate reductase, *folA* (reviewed in Andersson and Hughes 2010). Nalidixic acid resistance can also arise via upregulation of multi-drug efflux pumps. For consistency across antibiotics, we considered only resistance conferred by SNPs, which lead to excluding uncharacterised and efflux pump mutants from the data from Harmand et al. (2016). We analysed resistant mutants one mutational step away from a sensitive ancestor. Resistant strains had been generated either by mutant construction (*E. coli* K-12: rifampicin, trimethoprim) or fluctuation tests (*E. coli* K-12: nalidixic acid, *Pseudomonas*: rifampicin). Fitness had been measured using either growth rates or area-under-the-curve from growth curve data, or from competition experiments (shown to be equivalent by Vogwill and Maclean 2015). We calculated Pearson correlation coefficients for fitness for all pairs of antibiotic concentrations.

### Observed frequency of different resistance mutations

We obtained data on the frequency of occurrence of different resistance mutations prior to and following sustained selection for resistance from published evolution experiments (see Table S1). The studies characterised mutant identity either by Sanger sequencing known resistance regions, or by whole-genome resequencing. Frequency was scored as the presence of a given mutation among the replicate populations, divided by the total number of populations. Mutant frequency prior to antibiotic treatment had been quantified using fluctuation tests (Luria and Delbrück 1943), which identifies resistance mutations occurring in an initially sensitive populations by plating cultures on antibiotic-containing solid growth medium. Overnight growth cultures had been plated on agar containing rifampicin: *E. coli* K-12: 100 mg/L rifampicin (Garibyan et al. 2003, using only the data from the unmutagenized wild-type strain); *Pseudomonas aeruginosa* PAO1: 60 mg/L and *P. fluorescens* Pf0-1: 30 mg/L (Vogwill et al. 2014). The approach of relating fitness to frequency is conceptually similar to that used by MacLean et al. (2010) for secondary mutations arising in resistant genetic backgrounds. We associated mutant allele frequency with the fitness estimates described in the previous section.

For *E. coli* K-12 and *P. fluorescens* Pf0-1, mutation frequency following sustained antibiotic selection had been quantified using a selection experiment, which in both cases involved serial transfers in lysogeny broth (LB) containing rifampicin. *E. coli* K-12 populations were exposed to increasing concentrations of rifampicin (from 0 to 190 mg/L) over a period of approximately 160 generations (Lindsey et al. 2013). *P. fluorescens* Pf0-1 had been exposed to a constant concentration of rifampicin (6.4 mg/L, which inhibits growth rate of the sensitive strain to 10% relative to the antibiotic-free environment) for approximately 110 generations (Vogwill et al. 2016a).

### Simulation model for resistance evolution

Given that the nature of environmental pleiotropy can vary between strains and antibiotics, we simulated the evolution of resistance varying the correlation between fitness in both environments. We modelled growth in an initially antibiotic-free environment, followed by a sudden introduction of antibiotic at a sustained fully-inhibitory concentration. Populations initially consisted of *N*_0_(0) genetically identical individuals. We consider three scenarios for environmental pleiotropy: positively correlated, uncorrelated, and negatively correlated fitness.

A set of *S* = 50 fitness effects in each environment were drawn from a normal distribution with mean $\bar r_j$ and standard deviation $s_{r_j}$, where j refers to the environment (antibiotic-free or antibiotic-containing). The same set of fitness effects was used for each set of parameter values used in the simulation. The mean and standard deviation of fitness effects were estimated from the sample of growth rates of resistant *E. coli* K-12 mutants at 0 and 37.2 mg/L rifampicin (Lindsey et al. 2013). To simulate environmental pleiotropy, each mutation was ranked by fitness in the antibiotic-free environment, and then assigned either the same rank (positive pleiotropy), the opposite rank (negative pleiotropy) or a random rank (neutral pleiotropy) in the antibiotic-containing environment. We simulated differences in mutation rates of different loci, such that the sum of mutation rates of all loci was equal to the global mutation rate *μ* (estimated in Krašovec et al. 2014).

Population growth was modelled using a modified version of the deterministic discrete-time Lotka-Volterra competition model (Volterra 1926; Lotka 1932), with the addition of periodic reductions in population size (’bottlenecks’) and density-independent death. Variables and parameters are given in Table 1. Population growth rate was density-dependent, and total population size was limited by the population carrying capacity *K*. Density-independent death term *d* was included to simulate cell death due to environmental factors (e.g., antibiotic or immune system), and a random variable *M*_*i*_ for the number of allele *i* mutants arising among offspring produced by the wild-type, where *Mi*(*t* + 1) ~ Binomial(*μ*_*I*_, *N*_0_(*t* + 1)−*N*_0_(t)). To investigate the role of demographic history, we varied bottleneck size (*B*, the percentage of population surviving), and initial population size of the sensitive wild-type (*N*_0_(0)). Populations were subjected to a bottleneck every *b* generations, which reduced total population size to a percentage *B* of the original size. Any *N*_*i*_(*t*)<1 was considered to have gone extinct (i.e., *N*_*i*_ was set to 0). On the introduction of antibiotic at *t* = *t*_*A*_, the growth rates of all individuals switch from the antibiotic-free value to the antibiotic-containing value, including the ancestor, whose growth rate becomes zero (and hence *M*_*i*_ = 0 for all *t* > *t*_*A*_). Equation 1 describes the number of individuals of genotype *i*,

1 $$N_i\left( {t + 1} \right) = B^{\sigma \left( t \right)}\left( {N_i\left( t \right)\left[ {r_{ij}\left( {1 - \mathop {\sum}\limits_{i = 0}^S N_i\left( t \right)/K} \right) - d} \right] + M_i} \right),$$

where *σ*(*t*) is a switch denoting whether a bottleneck occurs at *t*,

$$\sigma \left( t \right) = \left\{ {\begin{array}{*{20}{c}} 0 & {{\mathrm{for}}\,{\it{t}}\,mod\,b > 0} \cr 1 & {{\mathrm{for}}\,{\it{t}}\,mod\,b = 0.} \end{array}} \right.$$

**Table 1:** List of variables and parameters for simulated resistance evolution

*Variables*		
*t*	Time (generations)	
*N*_*i*_(*t*)	Population size of mutant allele *i* at *t*	
*M*_*i*_(*t*)	New mutant *i* individuals arising by mutation at *t*	
*Population parameters*		Value(s)
*K*	Population carrying capacity	10^9^
*N*_0_(0)	Initial population size of sensitive	10^5^, 10^6^, 10^7^, 10^8^
*b*	Generations between bottlenecks	10
*d*	Death rate	0, 0.01, 0.02,…,0.1
*B*	Bottleneck size (% surviving)	0.1, 1, 10%
*t*_max_	Duration of simulated evolution (generations)	500
*t*_*A*_	Number of generations spent in antibiotic-free environment	100, 200, 300, 400
*Ancestor and resistant mutant parameters*		
*S*	Number of resistance mutations	50
*i*	Strain or mutation identifier	*i* = 0 (ancestor)
		1 ≤ *i* ≤ *S* (mutants)
$\bar r_f$	Antibiotic-free mean growth rate	1.1365239
$s_{r_f}$	Antibiotic-free standard deviation of growth rate	0.2161931
$\bar r_c$	Antibiotic-containing mean growth rate	0.9311666
$s_{r_c}$	Antibiotic-containing standard deviation of growth rate	0.2946277
$r_{ij}$	Growth rate of mutant *i* in environment *j*	${\mathrm{\sim Normal}}\,\left( {\mu = \bar r_j,\,\sigma = s_{r_j}} \right)$, for *i* ≠ 0
$r_{0_f}$	Sensitive ancestor growth rate (antibiotic-free)	2
$r_{0_c}$	Sensitive ancestor growth rate (antibiotic-containing)	0
*μ*	Global mutation rate to resistance	10^−8^
*μ*_*i*_	Frequency of occurrence of mutation *i*	~Uniform (0,*μ*/*S*), for *i* ≠ 0

Values for $\bar r_j$ and $s_{r_j}$ are estimated from rifampicin-resistant *E. coli* K-12 growth data at 0 and 37.2 mg/L rifampicin (Lindsey et al. 2013)*. μ* is estimated in Krašovec et al. (2014).

and *j* is an index describing the environment (‘antibiotic-free’ for *t* < *t*_A_ or’antibiotic containing’ for *t* ≥ *t*_A_).

For each combination of parameters, 100 replicate simulations were run for 500 generations of evolution. We scored mutant frequency as the proportion of the total population represented by that mutant across replicate simulations. Simulations and analyses were performed in R 3.4.2 (R Core Team 2017). Scripts are provided in the online supplementary material and a graphical representation of the pipeline is provided in Figure S1.

## Results

### Environmental pleiotropy among resistance alleles is neutral or positive for three antibiotics

**Fig. 1:**
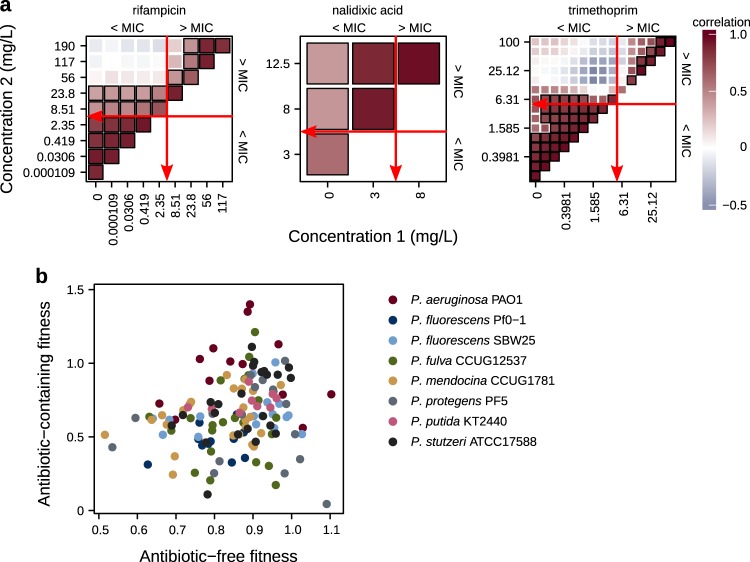
**a** Pearson correlation coefficients for fitness measured between pairs of antibiotic concentrations in antibiotic resistant *E. coli* K-12. black borders indicate significance at *p* < 0.05, red arrows indicate the minimum inhibitory concentration (MIC) of the sensitive ancestor. **b** Relationship between antibiotic-free and antibiotic-containing fitness in 8 rifampicin-resistant *Pseudomonas* species (60 mg/L for *P. aeruginosa* PAO1 and 30 mg/L for all others). Data sources given in Table S1.

Using previously published data (Table S1), we investigated the environmental pleiotropy of resistance mutations by correlating their fitness effects in the presence of different concentrations of antibiotic. In *E. coli* K-12 resistant to rifampicin, nalidixic acid, or trimethoprim, fitnesses that were either above or below the MIC of the sensitive ancestor were significantly positively correlated, but values on opposite sides of the MIC were typically not significantly correlated (Fig. 1a). Similarly, we found little correlation between fitness in the presence and absence of rifampicin among rifampicin-resistant strains from the genus *Pseudomonas* (Fig. 1b; Table 2). Therefore, although resistance alleles have negative pleiotropic effects relative to their sensitive ancestor (Melnyk et al. 2015; Vogwill and MacLean 2015), we have not found evidence of strong negative pleiotropy *among* resistance alleles.

**Table 2:** Spearman rank correlation between antibiotic-free and antibiotic-containing fitness in *Pseudomonas* species (shown in Fig. 1b).

Species	Estimate	*n*	Statistic	*p*-value
*P. aeruginosa* PAO1	0.0659	13	340	0.835
*P. fluorescens* Pf0-1	0.2364	11	168	0.485
*P. fluorescens* SBW25	0.3113	17	562	0.223
*P. fulva* CCUG12537	0.1518	25	1950	0.479
*P. mendocina* CCUG1781	0.1409	24	1980	0.511
*P. protegens* PF5	−0.0500	16	714	0.856
*P. putida* KT2440	0.4303	10	94	0.218
*P. stutzeri* ATCC17588	0.3934	23	1230	0.063

### Mutant allele frequency is associated with locus mutation rates, and fitness in antibiotic-containing environments

**Fig. 2:**
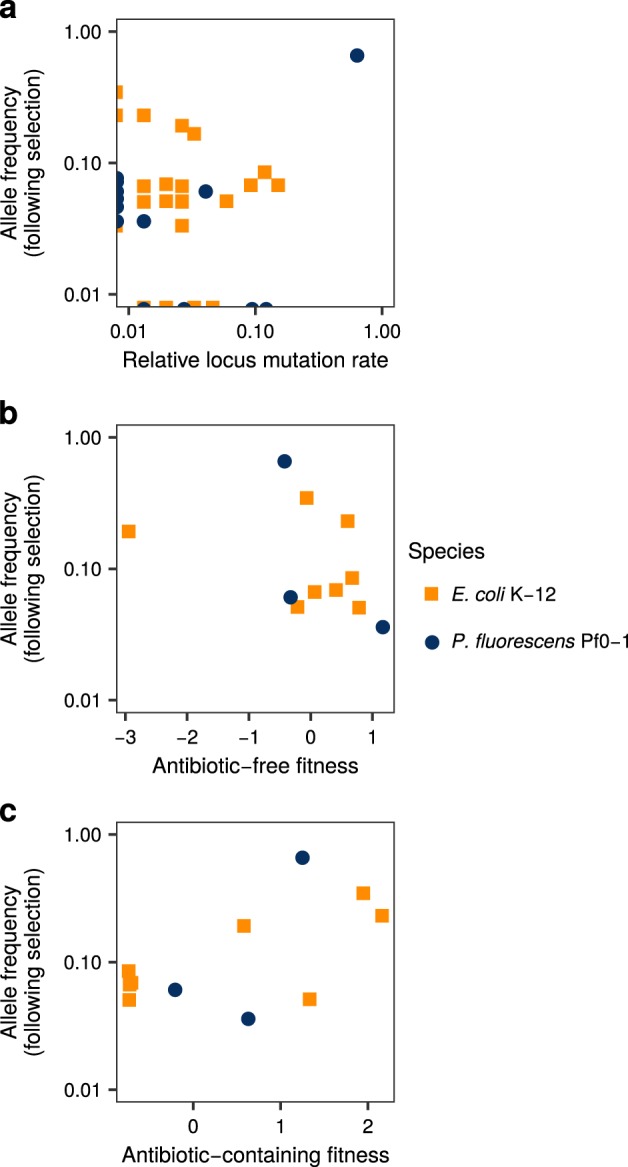
Frequency of rifampicin resistant mutants observed following selection for rifampicin resistance in *E. coli* K-12 and *P. fluorescens* Pf0-1, versus **a** frequency observed during fluctuation test in the absence of selection for resistance, **b** fitness in antibiotic-free environment, and **c** fitness in antibiotic-containing environment. (Fitness normalised to represent data on a common scale). Data sources given in Table S1.

Given that evolutionary rescue depends on the chance occurrence of resistance mutations, it is possible for the fitness of mutants to be disconnected from their frequency following sustained selection. Chance occurrence of mutations, driven by the mutation rate of different resistance loci, could in principle be a better predictor. The frequency with which different resistance alleles at different loci arise by mutation can vary considerably (e.g., Garibyan et al. 2003; Vogwill et al. 2014, as shown above). To test the role of such locus mutation rates, we compared how frequently different rifampicin resistant alleles in *E. coli* K-12 and *P. fluorescens* Pf0-1 were observed following acute versus sustained selection for resistance (i.e., lethal antibiotic selection following growth in an antibiotic-free environment in a fluctuation test, estimating the locus mutation rate, versus long-term selection in the presence of antibiotic). Frequency observed following sustained selection was positively associated with frequency during lethal selection (Fig. 2a, rank correlation *ρ* = 0.47, *S*_34_ = 4147, *p* = 0.004), suggesting selection on fitness does not completely overwhelm the signature of spontaneous mutation. However, this relationship was driven by high frequencies of a specific SNP at the same locus in both species (D516G in *E. coli* K-12, D521G in *P. fluorescens* Pf0-1). Eliminating this mutation, the association remains positive, but not significant (rank correlation *ρ* = 0.30, *S*_29_ = 3486, *p* = 0.10), demonstrating the importance of considering mutational hot-spots in predicting evolution (Orencia et al. 2001; Barlow and Hall 2002; Chattopadhyay et al. 2009).

The absence of a positive correlation between fitness under low and high antibiotic concentrations suggests that different mutants will be favoured in the presence and absence and of antibiotic. We therefore tested whether frequency following sustained selection is associated with fitness in either environment. We found opposing effects of fitness in each environment on mutant frequency (Steiger’s z-test for differences of correlations, *z* = 2:26, *p* = 0:024, Diedenhofen and Musch 2015). Frequency was negatively associated with fitness in the absence of antibiotic (Fig. 2b) and positively associated with fitness in the presence of antibiotic (Fig. 2c). These associations cannot be attributed to correlation between locus mutation rates and fitness (see Figure S2 and online supplementary text).

### Demography and pleiotropy dictate the effects of locus mutation rate and fitness on allele frequencies during simulated resistance evolution

**Fig. 3:**
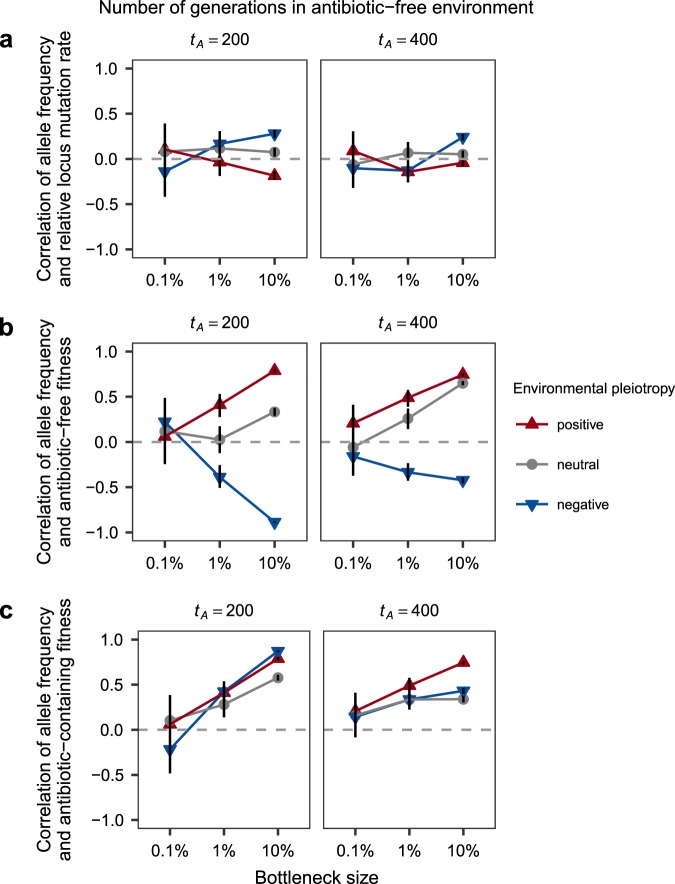
In simulations of sustained selection for antibiotic resistance, the effects of **a** locus mutation rate, **b** fitness in the antibiotic-free environment, and **c** antibiotic-containing fitness on resistance allele frequency varied under different scenarios for environmental pleiotropy. Data show the Pearson correlation across 100 replicate simulations. Error bars show the upper and lower 95% correlation coefficient confidence limits. Parameter values are given in Table 1 (Results for *t*_*A*_ = 200 or 400, *d* = 0 and *N*_0_(0) = 10^5^ shown. See Figures S4 and S5 for additional parameter value simulations.)

**Fig. 4:**
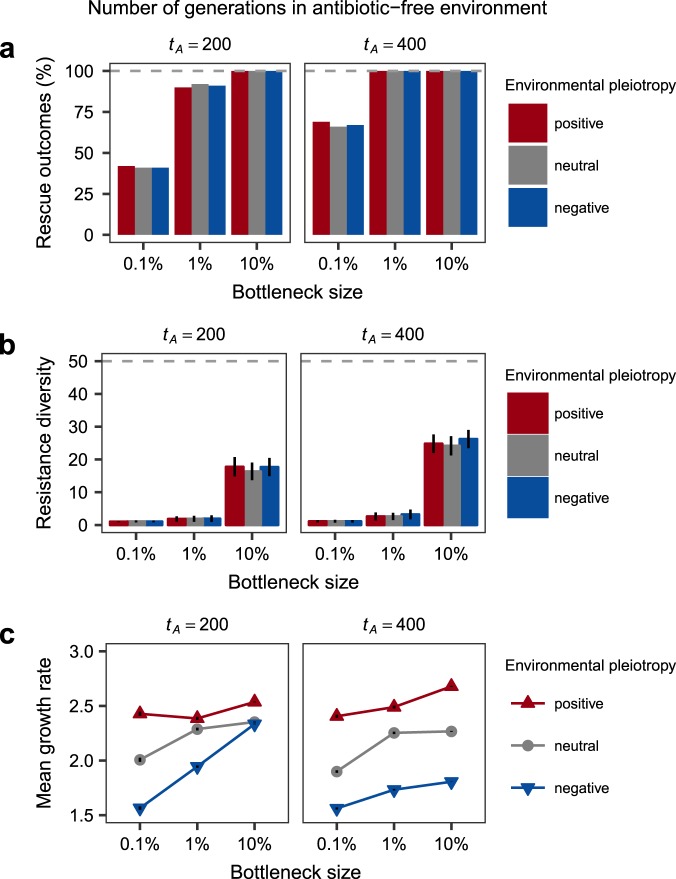
Simulated variation in demographic history (i.e., generations in antibiotic-free conditions and bottleneck size) and environmental pleiotropy influenced population-level adaptation to sustained antibiotic resistance selection. Evolutionary rescue (**a**) and mean resistance diversity (i.e., number of resistance alleles in the population, **b** were both influenced by demography, but not pleiotropy. Mean population growth rate (in the presence of antibiotic, **c** following simulated resistance evolution was influenced by both demography and pleiotropy. For **b** and **c**, error bars represent ±1 standard deviation. Parameter values are given in Table 1 (Results summarised over 100 replicate simulations for *t*_*A*_ = 200 or 400, *d* = 0 and *N*_0_(0) = 10^5^ shown. See Figures S6 and S7 for additional parameter value simulations.)

To investigate the effects of demographic history and environmental pleiotropy on resistance evolution, we simulated evolution in populations initially growing in an antibiotic-free environment, followed by a switch to an antibiotic-containing environment. Evolution was simulated for 500 generations, with periodic population bottlenecks (*B* = 10, 1, or 0.1% survival) occurring every *b* = 10 generations to simulate variation in demographic history. Each population was exposed to a ‘bacteriostatic’ antibiotic after a varying number of generations (although qualitatively similar results were obtained for a ‘bacteriocidal’ antibiotic, not shown). Resistance mutations had either positive, neutral, or negative correlations between fitness in antibiotic-free and antibioti c-containing environments. Each combination of parameters, given in Table 1 was simulated 100 times. Representative output from the simulation is shown in Figure S3. The main text Figs. 3 and 4 show results for antibiotic introduction times of *t*_*A*_ = 200 or 400 generations (out of 500), no death (*d* = 0), and an initial population size of *N*_0_(0) = 10^5^. Results for other parameter values are shown in Figures S4–S7.

The effects of locus mutation rate and fitness in each environment on allele frequency following sustained selection is shown in Fig. 3. Figure 3a shows the correlation between locus mutation rate and the frequency of each mutant observed following sustained selection. As in the experimental data, the association between allele frequency and locus mutation rate was weak overall, though strongest for the largest bottleneck size considered (*B* = 10%) under negative pleiotropy. Figure 3b shows the effect of fitness in the antibiotic-free environment is contingent on both demography and pleiotropy. At the *B* = 0.1% bottleneck size, the effects of pleiotropy are negligible, with fitness only weakly associated with frequency. For larger bottleneck sizes (*B* = 1 and 10%), positive and neutral pleiotropy demonstrate similar relationships between frequency and fitness. In contrast, under negative pleiotropy, this correlation decreased from positive to negative as bottleneck size increased, except when populations spent most of their time in the antibiotic-free environment. Figure 3c shows the effect of fitness in the antibiotic-containing environment was again contingent on demographic history, but less dependent on environmental pleiotropy.

### Effects of demography and pleiotropy on population rescue, diversity, and mean fitness in simulated populations

The inability of the ancestor to reproduce in the presence of antibiotic makes evolutionary rescue an uncertain outcome. The probability of evolutionary rescue was dependent on demographic history-population bottlenecks and generations spent in the antibiotic-free environment, but not environmental pleiotropy (Fig. 4a). Similarly, Fig. 4b shows that demographic history, but not pleiotropy, influenced the amount of resistance diversity within surviving populations. In contrast, Fig. 4c shows that both demographic history and environmental pleiotropy influence evolved population mean fitness in the antibiotic-containing environment (growth rate of resistance alleles is used as a proxy for absolute fitness as the ancestor cannot grow in the presence of antibiotic). Positive pleiotropy consistently resulted in the highest mean growth rates. Neutral pleiotropy gave intermediate mean growth rates, and negative pleiotropy the lowest mean growth rates. The effect of negative pleiotropy relative to neutral pleiotropy was largely overcome by large population bottlenecks, except for populations spending the bulk of time under antibiotic-free conditions. Despite diversity in such populations being high, selection has too few generations to overcome opposing selection in the antibiotic-free environment (Fig. 4 and Figure S4-5).

## Discussion

Survival of populations exposed to inhibitory antibiotic concentrations relies on the presence of costly resistance variation prior to treatment, and is a key example of evolutionary rescue. Selection thus operates in two environments, i.e., the presence and absence of antibiotic. Therefore, the association of fitness in each environment among resistance alleles (i.e., their environmental pleiotropy) may determine which alleles are ultimately favoured by selection. Previous work has characterised the ‘cost of resistance’ (Vogwill and Maclean 2015; Melnyk et al. 2015)—that is, negative pleiotropy between the fitness of a resistant mutant relative to its sensitive ancestor in the presence and absence of antibiotic. However, this says nothing about what determines the relative frequencies of different resistance alleles after antibiotic treatment. Bringing together published data and simulations, we investigated the nature and effects of environmental pleiotropy. Reanalysing fitness effects of resistance mutations suggests that pleiotropy varies over antibiotic concentrations (Fig. 1). We found no evidence for strong negative pleiotropy across resistance alleles between fitness in antibiotic-free and antibiotic-containing environments. When both antibiotic concentrations were either above or below the minimum inhibitory concentration (MIC) for their ancestor, fitness of different resistant alleles tended to be positively correlated (i.e., to show positive pleiotropy). However, fitness was typically uncorrelated (i.e., showing neutral pleiotropy) when comparing antibiotic concentrations on either side of the MIC (Fig. 1, data from Lindsey et al. 2013; Vogwill et al. 2014; Palmer et al. 2015; Vogwill 2016a; Harmand et al. 2016). This lack of a correlation for fitness between high and low antibiotic environments, evidence for neutral pleiotropy, suggests that selection will favour different mutations in each environment.

To test the prediction that environmental pleiotropy influences which resistance alleles are favoured under sustained antibiotic selection, we looked at how resistance allele frequencies associated with locus mutation rates and fitness in the presence and absence of antibiotic. In both empirical and simulated resistance evolution, allele frequency following sustained selection for resistance was weakly correlated with locus mutation rate (Figs. 2a, 3a), suggesting that both locus mutation rates and selection on fitness have a role in determining allele frequencies. Empirical mutant allele frequencies (from Lindsey et al. 2013; Vogwill et al. 2016b) showed opposing correlations with their fitness in antibiotic-free versus antibiotic-containing conditions (Fig. 2b, c). These results most closely matched the simulations for neutral and negative pleiotropy (Fig. 3b, c), which is consistent with the nature of pleiotropy found for the fitness effects of these alleles (Fig. 1). Notably, neither locus mutation rate, nor fitness in either environment, was a good predictor of resistance allele frequencies for populations put through the smallest bottlenecks (Fig. 3), due to the presence of only a single allele in each surviving population. These results highlight the difficulty in predicting evolution under environmental change, even if every characteristic of each mutation is known with perfect precision.

Although environmental pleiotropy can instigate evolutionary conflicts during resistance evolution, our results show that this effect is contingent on the demographic history of the population. Simulations allowed us to explore the interaction of environmental pleiotropy with the size of recurring population bottlenecks and time spent in antibiotic-free conditions. Correlations between allele frequencies and fitness in either environment were stronger for larger bottleneck sizes (Fig. 3b, c). This likely resulted from the association between bottleneck size and diversity that has been previously established (Nei et al. 1975). Consistent with previous work on evolutionary rescue (Lande 1988; Iwasa et al. 2004; Bell and Gonzalez 2009; Martin et al. 2013; Ramsayer et al. 2013; Bell 2013; Orr and Unckless 2014; Bell 2017), we found a higher probability of rescue, greater genetic diversity, and higher mean fitness in simulated populations subjected to weaker population bottlenecks (Fig. 4). Our work shows that environmental pleiotropy had no additional effect on the chance of evolutionary rescue or diversity, but did influence population mean fitness.

As our focus was on how locus mutation rates and fitness influence resistance allele frequencies, we consider only genetic diversity occurring at resistance loci (an approach used by others, e.g., Orr and Unckless 2014). Long-term evolution experiments suggest that this is likely an oversimplification, as beneficial mutations continue to accrue even after extensive periods of adaptation (see e.g., Good et al. 2017). The sweep of a non-resistance conferring beneficial mutations will reduce the overall diversity of resistance mutations in the ancestral genetic background. Consequently, the simulation may overestimate expected resistance allele diversity. Nonetheless, the general patterns associated with demographic history, namely that higher levels of diversity result in stronger effects of fitness, are likely to hold. Similarly, genetic variation at loci not considered by the model may affect the nature of environmental pleiotropy. Firstly, costs of resistance are lower in fitter ancestral backgrounds (Angst and Hall 2013), therefore variation at non-resistance loci that affect ancestral fitness may reduce the strength of negative pleiotropy. Secondly, compensatory mutations that arise in resistant genetic backgrounds mitigate the fitness costs of resistance in the absence of antibiotic, but do not necessarily increase fitness in its presence (Schulz zur Wiesch et al. 2010). Large-effect compensatory mutations tend to occur more frequently in high-cost resistance backgrounds (e.g., Barrick et al. 2010; MacLean et al. 2010; Moura de Sousa et al. 2015; Couce and Tenaillon 2015), also reducing the strength of negative pleiotropy. Ultimately, the extreme form of negative pleiotropy investigated in the simulations may not be a general feature of resistance evolution, consistent with the absence of strong negative pleiotropy in Fig. 1.

The majority of work on environmental pleiotropy has been performed in in vitro systems, but whether pleiotropy plays a critical role in in vivo resistance evolution remains to be determined. Resistance mutations are known to be costly when measured in hosts (Andersson and Hughes 2010), but environmental pleiotropy for resistance in variable environments is difficult to characterise, even in well-defined systems (Hall 2013; Maharjan and Ferenci 2017). The diversity of resistance mechanisms is also a challenge, as each may exhibit different pleiotropic effects (e.g., efflux pumps versus SNPs in Harmand et al. 2016. Resistance mechanisms induced by antibiotic exposure (e.g., beta-lactamase production) may be partially shielded from selection in the absence of antibiotic (Foucault et al. 2010). Further, plasmid-borne resistance genes, a major contributor to clinical resistance, typically bear a lower cost of resistance than chromosomal resistance mutations (Vogwill and MacLean 2015) and generally confer higher fitness in the presence of antibiotic (Uhlin and Nordström 1977). Plasmid carriage of a resistance gene is also associated with higher evolutionary potential for resistance (San Millan et al. 2017), due to higher mutational supply afforded by elevated gene copy number (Andersson and Hughes 2009; Sano et al. 2014). Determining whether the processes modelled here could constrain resistance evolution in clinically-relevant bacteria is an avenue for future research.

Our simulations suggest that a key component to predicting resistance evolution will be quantifying resistance diversity. Several factors are likely to contribute to the emergence of diversity during infection. Environmental-, community-associated or hospital-associated infectious populations will harbour different levels of diversity (Allen et al. 2010). Changes in life history traits during adaptation to the host environment, such as switching from commensal to pathogen, are associated with changes in diversity on a genomic scale (Golubchik et al. 2013; Young et al. 2017). As different forms of treatment result in different rates of resistance emergence (Felton et al. 2013), treatment regime will also dictate levels of resistance diversity. Resistance diversity will likely be greater when the rate of concentration increase is slower, as demonstrated by Lindsey et al. (2013). However, the rate of increase typical of laboratory evolution experiments may not accurately reflect that in vivo, as pharmacokinetic studies indicate concentrations change much faster during treatment (Kiang et al. 2014). This rapid increase suggests that, unlike in in vitro studies, the potential for in vivo resistance diversity to arise during antibiotic therapy may be limited. As our simulations showed the effects of fitness were dependent on diversity, fitness in clinical settings may thus have limited impact on resistance allele frequencies. Ultimately, predicting resistance evolution in wild microbial populations will require understanding both environmental pleiotropy and demographic history, each of which is a considerable logistical challenge.

### Data archiving

Data and analysis code are archived with Dryad (10.5061/dryad.gp5g0j0).

## Electronic supplementary material

Supplemental text and figures

Data and analysis code for empirical figures

Simulation code
