# Supplementary material for: Environmental pleiotropy and demographic history direct adaptation under antibiotic selection
Source: Heredity (Edinb). 2018 Sep 6;121(5):438–48. doi: 10.1038/s41437-018-0137-3 (PMC6180006; doi:10.1038/s41437-018-0137-3)
Supplement: Supplementary file 1 — Supplemental text and figures [file 41437_2018_137_MOESM1_ESM.pdf]

Supplemental Material: Environmental pleiotropy and demographic history direct adaptation under antibiotic selection

Danna R. Gifford[1,2], Rok Krašovec[1], Elizabeth Aston[3], Roman V. Belavkin[4], Alastair Channon[3], and Christopher G. Knight[2]

July 17, 2018

1. School of Biology, Faculty of Biology, Medicine and Health, The University of Manchester, Manchester, United Kingdom
2. School of Earth and Environmental Sciences, Faculty of Science and Engineering, The University of Manchester, Manchester, United Kingdom
3. School of Computing and Mathematics, Faculty of Natural Sciences, Keele University, Keele, United Kingdom
4. School of Science and Technology, Middlesex University, London, United Kingdom

Corresponding author Danna R Gifford (danna.gifford@manchester.ac.uk)

Address The University of Manchester, Michael Smith Building, Oxford Road, Manchester M13 9PT, United Kingdom, +44 7534715187

Supplementary information

Frequency of mutations prior to selection

To rule out methodological biases in the ability to detect resistant mutants, we determined whether mutant frequencies in the absence of selection (i.e. resulting from fluctuation tests) were associated either with the underlying mutational spectrum or fitness. Using mutation accumulation data for *E. coli* K-12 (Lee *et al.*, 2012) and *P. aeruginosa* PAO1 (Dettman *et al.*, 2016), we estimated the underlying mutational spectrum as the substitution rate of transitions (AT>GC and GC>TA) and transversions (AT>CG, AT>TA, GC>CG, and GC>TA). We used the mutational spectrum to calculate expected frequency for each type of substitution as the substitution rate estimated from mutation accumulation times the number of known resistance mutations of that type in *rpoB* (using all observed resistance mutations in all strains and treatments as known resistance loci, Garibyan *et al.*, 2003; Vogwill *et al.*, 2016a). We compared this expected frequency with the frequencies of each substitution type observed via fluctuation tests. We found a positive, though non-significant correlation between the underlying mutational spectrum and mutant frequency observed during fluctuation test (Spearman rank correlation, $S_{10} = 122.71$, $\rho = 0.57$, $p = 0.053$, Figure S2a). The frequency of mutations observed prior to selection was not significantly correlated with fitness effects in either antibiotic-free (Figure S2b, Spearman rank correlation, $S_{10} = 328.74$, $\rho = -0.49$, $p = 0.12$) or antibiotic-containing environments (Figure S2c, Spearman rank correlation, $S_{10} = 309.61$, $\rho = -0.40$, $p = 0.21$).

Table S1: Literature used for empirical fitness and frequency of occurrence correlations

Reference	Experimental method	Data description	Used in
DETTMAN *et al.* (2016)	mutation accumulation	Mutational spectrum in *P. aeruginosa* PAO1	Figure S2
GARIBYAN *et al.* (2003)	fluctuation test	Rifampicin resistance locus mutation rates in *E. coli* K-12	Figures 2 & S2
HARMAND *et al.* (2016)	fluctuation test	Fitness of nalidixic acid resistance mutations in *E. coli* K-12	Figure 1
LEE *et al.* (2012)	mutation accumulation	Mutational spectrum in *E. coli* K-12	Figure S2
LINDSEY *et al.* (2013)	selection experiment, mutant construction	Fitness and observed allele frequency after sustained rifampicin selection in *E. coli* K-12	Figures 1 & 2, simluation parameters
PALMER *et al.* (2015)	mutant construction	Fitness of trimethoprim resistance mutations in *E. coli* K-12	Figure 1
VOGWILL *et al.* (2014)	fluctuation test	Rifampicin resistance locus mutation rates in *Pseudomonas* species	Figures 2 & S2
VOGWILL *et al.* (2016a)	fitness assay	Fitness of rifampicin resistance mutations in *Pseudomonas* species	Figures 1 & 2
VOGWILL *et al.* (2016b)	selection experiment	Observed allele frequency after sustained rifampicin selection in in *P. fluorescens* Pf0-1	Figure 2

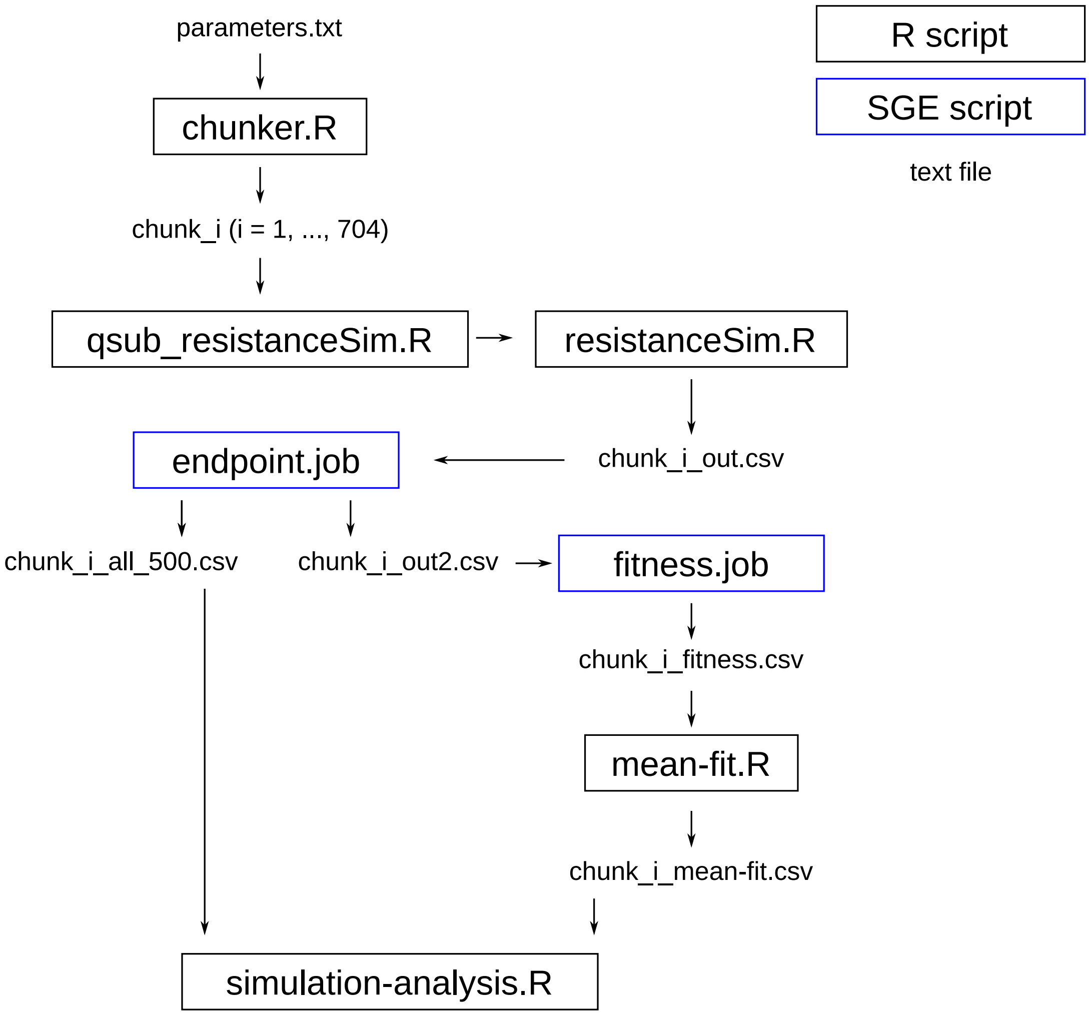

Figure S1: Graphical representation of simulation analysis pipeline.

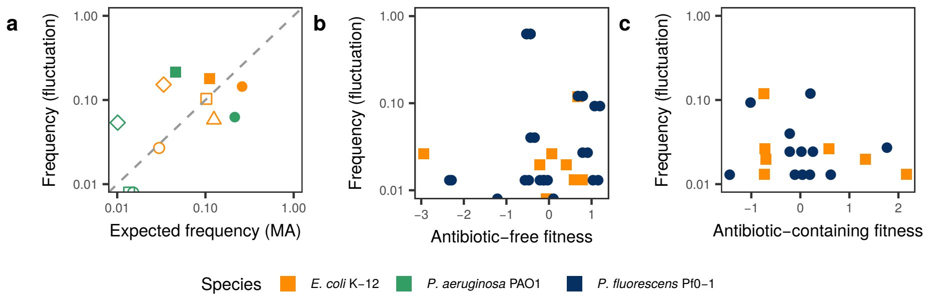

Figure S2: a Expected mutant frequency estimated from the underlying mutational spectrum versus mutant frequency prior to antibiotic exposure, showing transitions (■: AT>GC, ●: GC>TA) and transversions (□: AT>CG, ◇: AT>TA, ○ GC>CG, △: GC>TA). Relationship between mutant frequency and fitness: mutant frequency observed through fluctuation tests was not correlated with fitness in either **b** the antibiotic-free environment, or **c** antibiotic-containing environment. (Fitness was normalised to visualise data on a common scale. Data sources given in Table S1.)

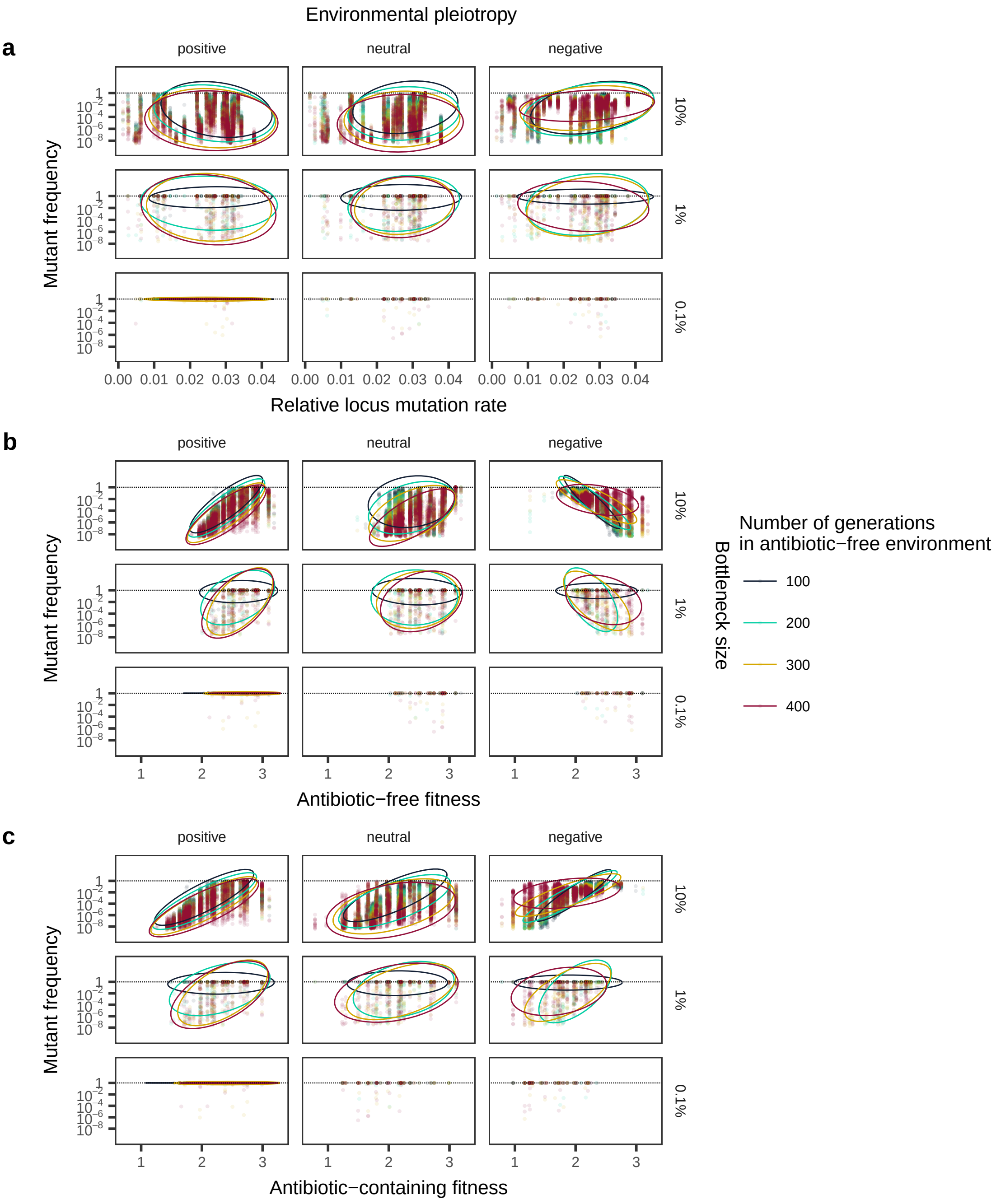

Figure S3: Representative output from simulation, showing the association between allele frequency and locus mutation rates (**a**), antibiotic-free fitness (**b**), and antibiotic-containing fitness (**c**). Ellipses represent 95% confidence level (not drawn when variance in mutant frequency was zero). Parameter values are given in Table 1 in the main text (Results for $t_A = 100, 200, 300, 400$, $d = 0$ and $N_0(0) = 10^5$ shown.)

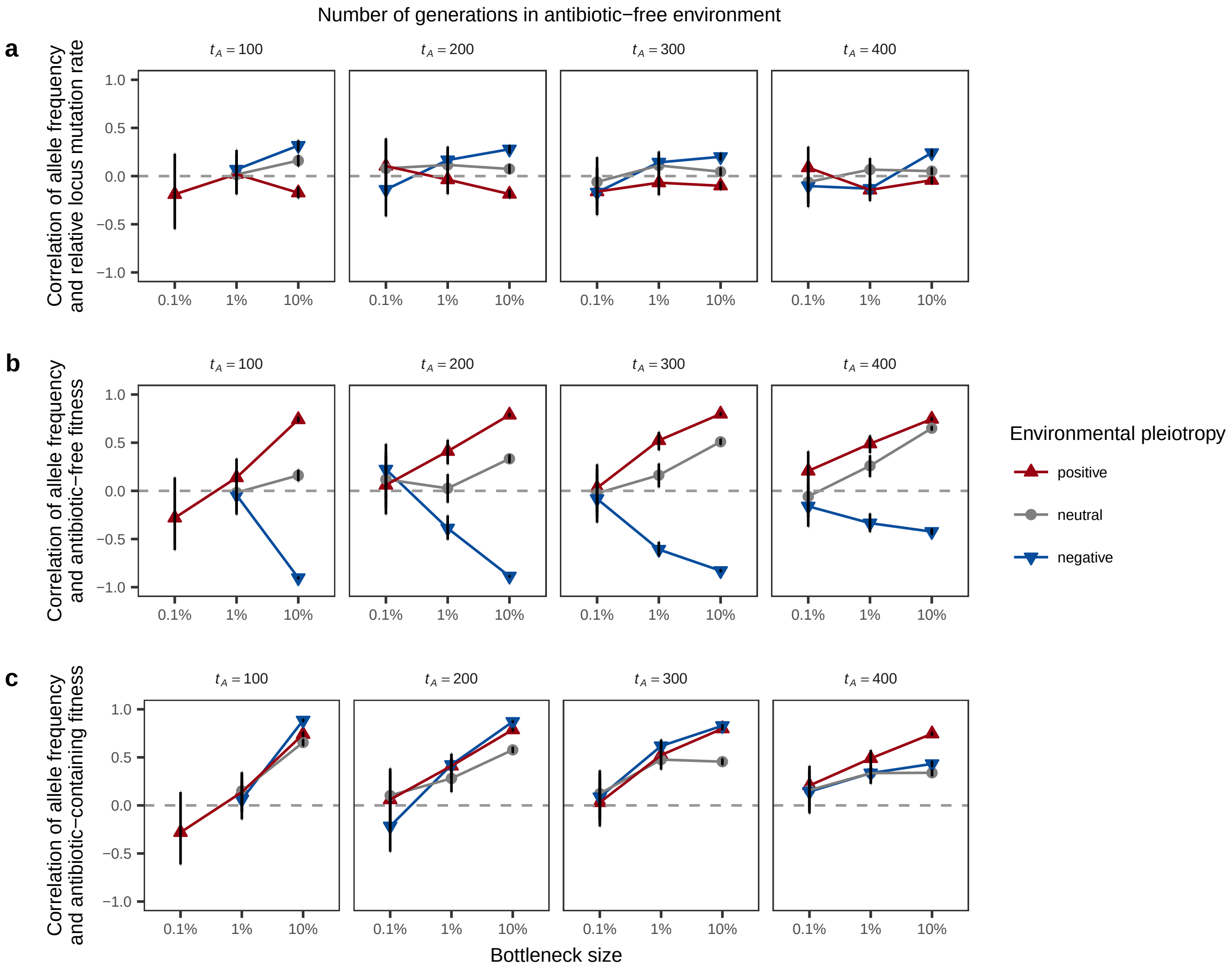

Figure S4: In simulations of sustained selection for antibiotic resistance, the effects of (**a**) locus mutation rate, (**b**) antibiotic-free fitness, and (**c**) antibiotic-containing fitness on resistance allele frequency varied under different scenarios for environmental pleiotropy. Data show the Pearson correlation across 100 replicate simulations. Error bars show the upper and lower 95% correlation coefficient confidence limits. (Results for $t_A = 100, 200, 300, 400$, $d = 0$ and $N_0(0) = 10^5$ shown. Other parameter values given in Table 1 in the main text.)

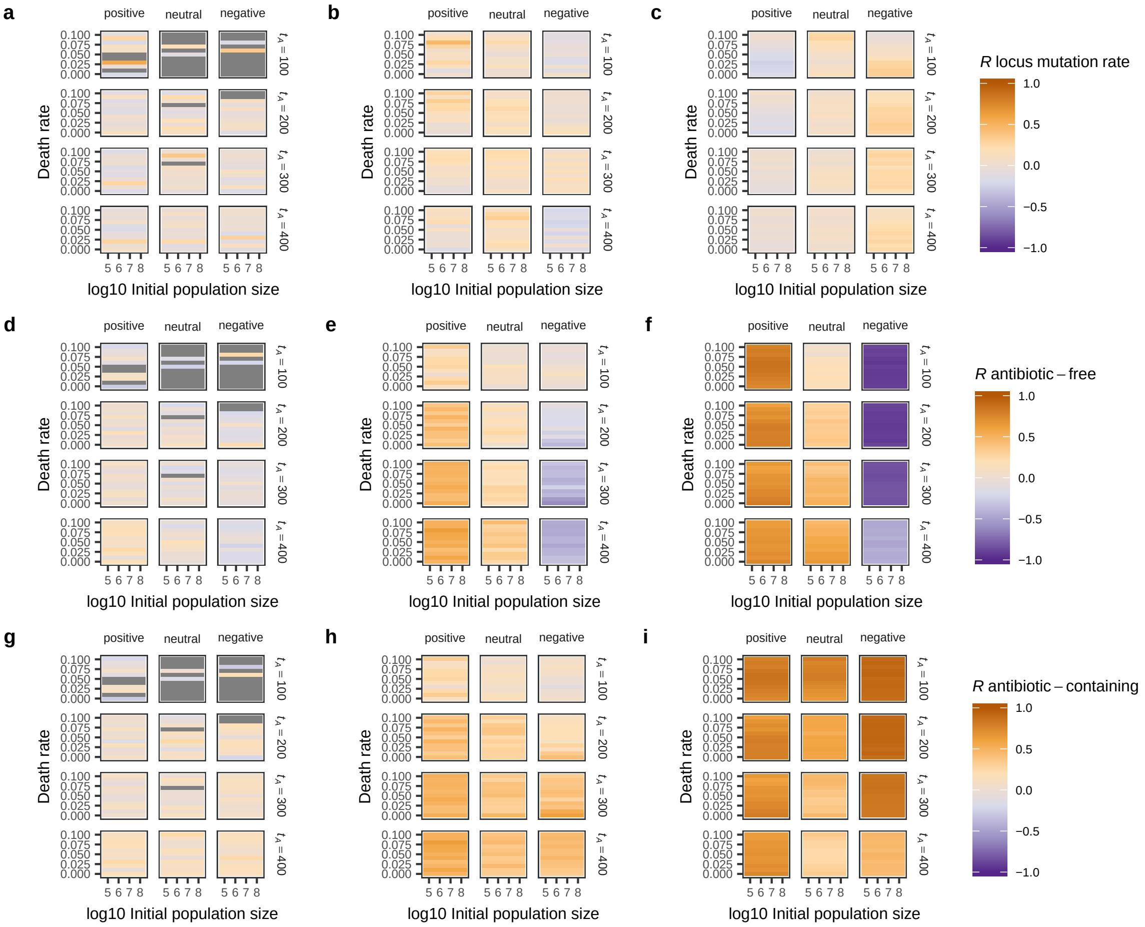

Figure S5: Simulated variation in death rates and initial population size following sustained selection for antibiotic resistance. Effects of locus mutation rate (**a-c**), resistant mutant fitness in antibiotic-free (**d-f**), and antibiotic-containing (**g-i**) environments on resistance allele frequency remain largely consistent across variation in death rate and initial population size. Parameter values given in Table 1 in the main text.

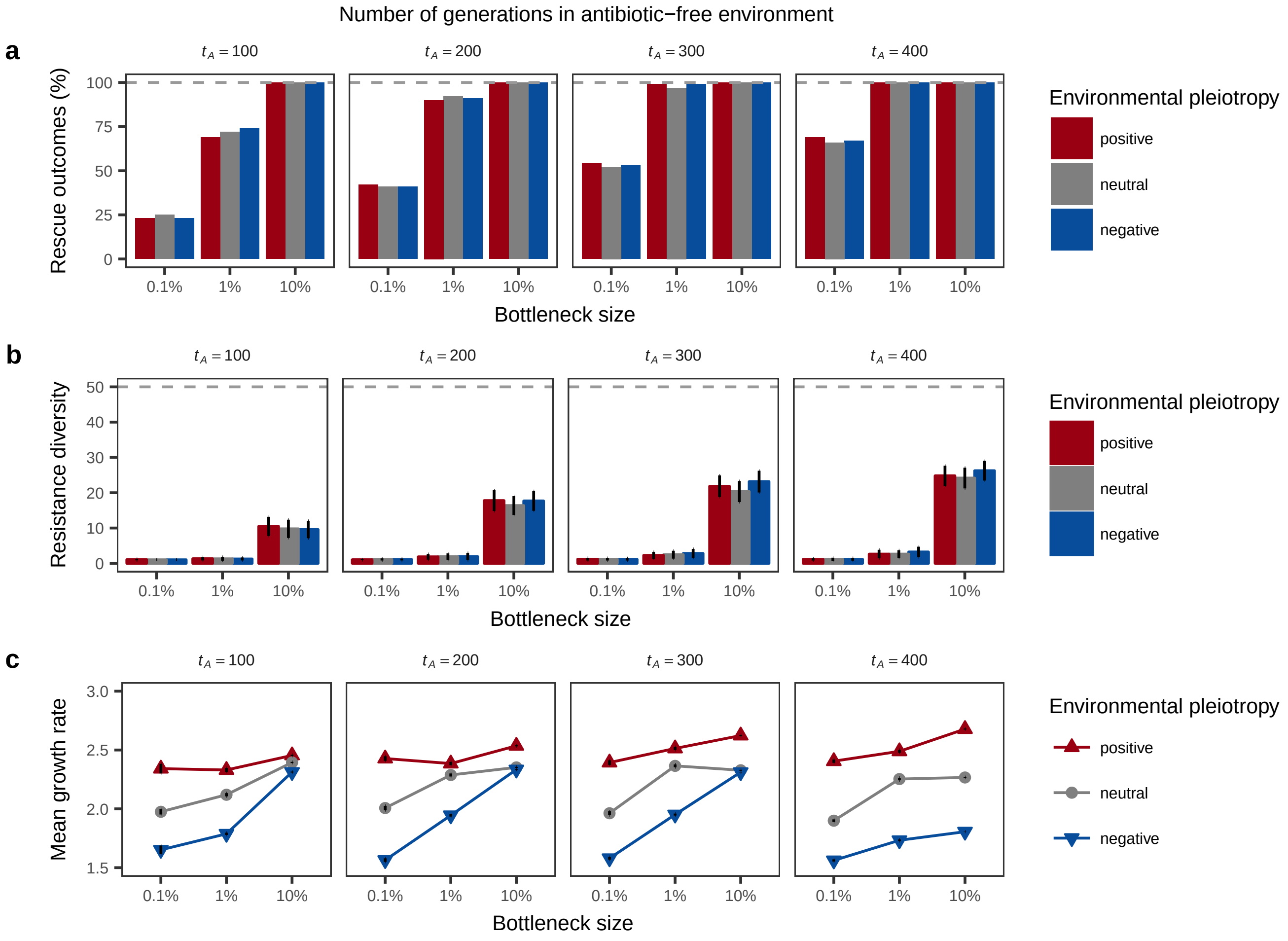

Figure S6: Simulated variation in demographic history (i.e. generations in antibiotic-free conditions and bottleneck size) and environmental pleiotropy influenced population-level adaptation to sustained antibiotic resistance selection. Evolutionary rescue (**a**) and mean resistance diversity (i.e. number of resistance alleles in the population, **b**) were both influenced by demography, but not pleiotropy. Mean population growth rate (in the presence of antibiotic, **c**) following simulated resistance evolution was influenced by both demography and pleiotropy. For b and c, error bars represent ± 1 standard deviation. Parameter values are given in Table 1 in the main text (Results for $t_A = 100$, 200, 300, 400, $d = 0$ and $N_0(0) = 10^5$ shown.)

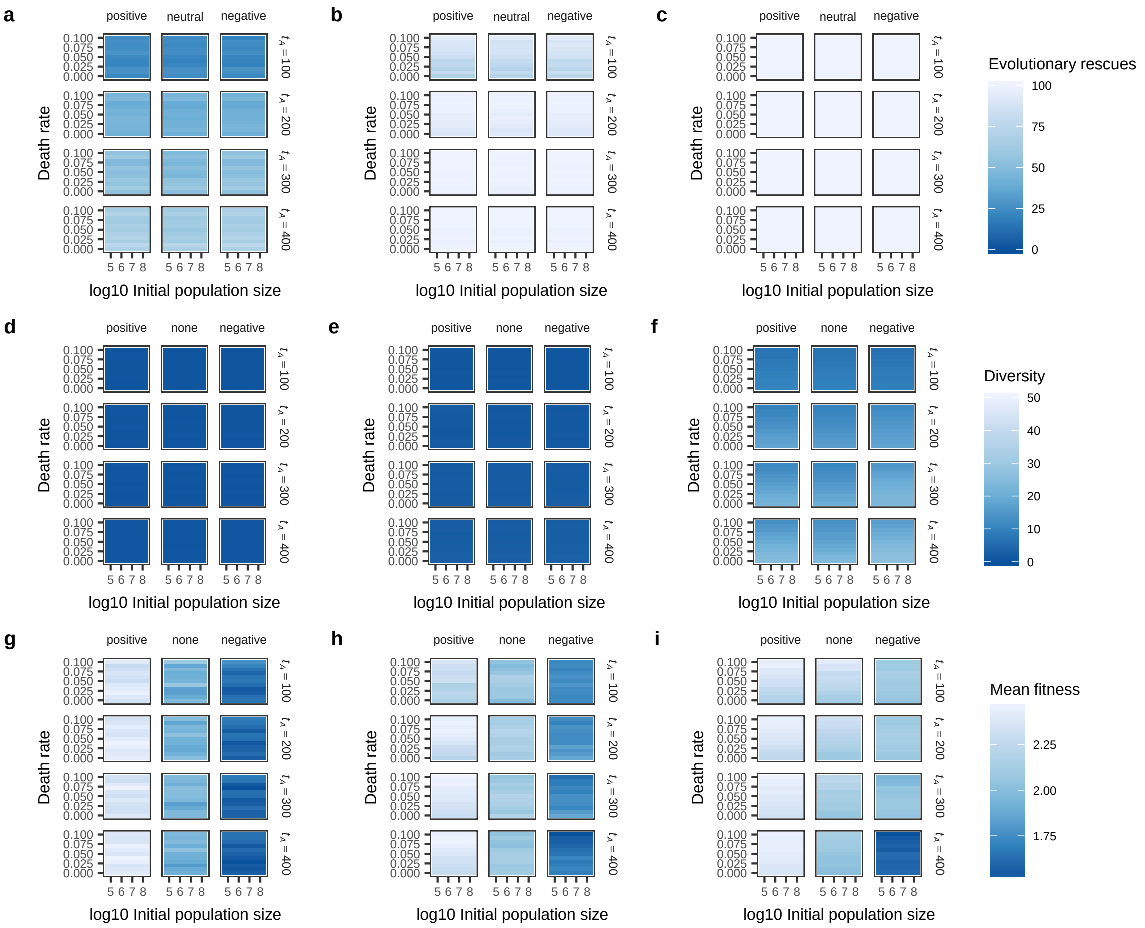

Figure S7: Simulated variation in death rates and initial population size following sustained selection for antibiotic resistance. Effects of demographic history and environmental pleiotropy on evolutionary rescue (**a-c**, resistance diversity (**d-f**), and mean fitness (**g-i**) remain largely consistent across variation in death rate and initial population size, following resistance evolution simulated for 500 generations for 100 replicate simulations. Parameter values given in Table 1 in the main text.

References

DETTMAN, J. R., J. L. SZTEPANACZ, and R. KASSEN, 2016 The properties of spontaneous mutations in the opportunistic pathogen *Pseudomonas aeruginosa*. BMC Genomics **17**.

GARIBYAN, L., T. HUANG, M. KIM, E. WOLFF, A. NGUYEN, *et al.*, 2003 Use of the *rpoB* gene to determine the specificity of base substitution mutations on the *Escherichia coli* chromosome. DNA Repair **2**: 593–608.

HARMAND, N., R. GALLET, R. JABBOUR-ZAHAB, G. MARTIN, and T. LENORMAND, 2016 Fisher's geometrical model and the mutational patterns of antibiotic resistance across dose gradients. Evolution **71**: 23–37.

LEE, H., E. POPODI, H. TANG, and P. L. FOSTER, 2012 Rate and molecular spectrum of spontaneous mutations in the bacterium*Escherichia coli* as determined by whole-genome sequencing. Proceedings of the National Academy of Sciences **109**: E2774–E2783.

LINDSEY, H. A., J. GALLIE, S. TAYLOR, and B. KERR, 2013 Evolutionary rescue from extinction is contingent on a lower rate of environmental change. Nature **494**: 463–467.

PALMER, A. C., E. TOPRAK, M. BAYM, S. KIM, A. VERES, *et al.*, 2015 Delayed commitment to evolutionary fate in antibiotic resistance fitness landscapes. Nature Communications **6**: 7385.

VOGWILL, T., M. KOJADINOVIC, V. FURIÓ, and R. C. MACLEAN, 2014 Testing the role of genetic background in parallel evolution using the comparative experimental evolution of antibiotic resistance. Molecular Biology and Evolution **31**: 3314–3323.

VOGWILL, T., M. KOJADINOVIC, and R. C. MACLEAN, 2016a Epistasis between antibiotic resistance mutations and genetic background shape the fitness effect of resistance

across species of *Pseudomonas*. Proceedings of the Royal Society B: Biological Sciences **283**: 20160151.

VOGWILL, T., R. L. PHILLIPS, D. R. GIFFORD, and R. C. MACLEAN, 2016b Divergent evolution peaks under intermediate population bottlenecks during bacterial experimental evolution. Proceedings of the Royal Society B: Biological Sciences **283**: 20160749.
