# Supplementary material for: Environmental pleiotropy and demographic history direct adaptation under antibiotic selection
Source: Heredity (Edinb). 2018 Sep 6;121(5):438–48. doi: 10.1038/s41437-018-0137-3 (PMC6180006; doi:10.1038/s41437-018-0137-3)
Supplement: Supplementary file 3 — Simulation code [file 41437_2018_137_MOESM3_ESM.zip › simulation/pipeline.pdf]

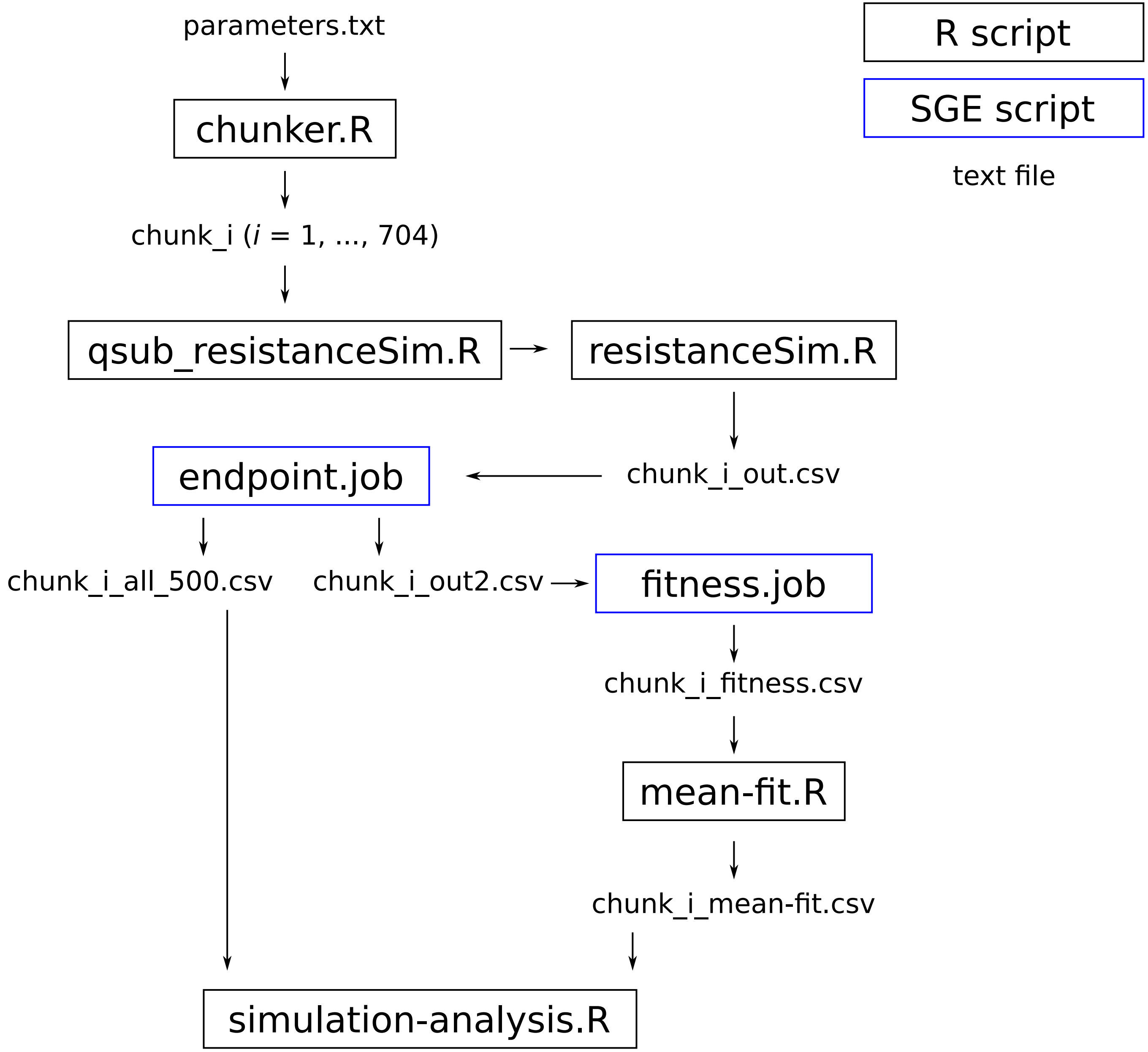
parameters.txt
chunker.R
chunk_i (i = 1, ..., 704)
qsub_resistanceSim.R
resistanceSim.R
chunk_i_out.csv
endpoint.job
chunk_i_all_500.csv
chunk_i_out2.csv
fitness.job
chunk_i_fitness.csv
mean-fit.R
chunk_i_mean-fit.csv
simulation-analysis.R
R script
SGE script
text file
